# A cryptic role for reciprocal helping in a cooperatively breeding bird

**DOI:** 10.1038/s41586-025-08958-4

**Published:** 2025-05-07

**Authors:** Alexis D. Earl, Gerald G. Carter, Arden G. Berlinger, Elkana Korir, Shailee S. Shah, Wilson N. Watetu, Dustin R. Rubenstein

**Affiliations:** 1 Department of Ecology, Evolution, and Environmental Biology, Columbia University, New York, NY USA; 2 Mpala Research Centre, Nanyuki, Kenya; 3 Department of Ecology and Evolutionary Biology, Princeton University, Princeton, NJ USA; 4 Smithsonian Tropical Research Institute, Panama City, Republic of Panama; 5 Howard Hughes Medical Institute, Chevy Chase, MD USA; 6 Present Address: Department of Computational Biology, Cornell University, Ithaca, NY USA; 7 Present Address: Department of Plant Sciences, University of Cambridge, Cambridge, UK; 8 Present Address: Cornell Lab of Ornithology, Cornell University, Ithaca, NY USA

**Keywords:** Social evolution, Evolutionary ecology

## Abstract

Identifying the mechanisms that underlie cooperation is fundamental to biology^1^. The most complex form of cooperation in vertebrates occurs in cooperative breeders, in which helpers forego reproduction and assist in raising the young of others, typically relatives^2^. Not all cooperative societies, however, are kin-based—nearly half of all avian^3^ and mammalian^4^ cooperative breeders form mixed-kin societies, much like those of humans^5^. Kin selection in mixed-kin societies occurs when individuals gain indirect fitness from the preferential helping of relatives^6^, but helpers also frequently assist non-kin^7^, highlighting a potential role for direct fitness in stabilizing cooperative societies^7,8^. Here, using a 20-year study of superb starlings (*Lamprotornis superbus*), we examined how direct and indirect fitness jointly influence helping behaviour. Although we detected kin-biased helping (demonstrating kin selection), non-kin helping was common despite opportunities to aid kin. Unexpectedly, specific pairs maintained long-term reciprocal helping relationships by swapping social roles across their lifetimes—a subtle pattern of reciprocity requiring decades of observation to detect. Given the frequency of non-kin helping and the occurrence of reciprocal helping among both kin and non-kin, helping behaviour in superb starlings seems to be greatly influenced by direct fitness. However, the relative importance of direct and indirect fitness varied with helpers’ sex and dispersal history. By uncovering a cryptic yet crucial role of long-term reciprocal helping, we suggest that reciprocity may be an underappreciated mechanism promoting the stability of cooperatively breeding societies.

## Abstract (web summary)

A study of the cooperative breeding behaviour of superb starlings during 40 consecutive breeding seasons over 20 years reveals long-term reciprocal helping between both related and unrelated individuals.

## Main

Beyond the potential indirect fitness benefits of aiding relatives (kin selection), helping others—whether kin or non-kin—can yield long-term benefits for lifetime direct fitness. Evolutionary models of cooperation for direct fitness show that selection can favour helping when it increases either by-product benefits (pseudo-reciprocity) or reciprocal help (reciprocity)^9–12^. By-product benefits and reciprocal helping can also coexist at intermediate levels when animals form long-term cooperative relationships^13–16^. Regardless of the underlying mechanisms, the direct fitness benefits of enabling or promoting reciprocal help from kin and non-kin can make greater contributions to inclusive fitness than the indirect fitness benefits of altruistically helping kin^17,18^. Yet, empirical studies highlighting the role of reciprocal help in the evolution of helping behaviour in cooperatively breeding birds^19,20^ and mammals^21^ are rare. Moreover, assessing the relative importance of direct versus indirect fitness for the evolution of helping has proved challenging. One difficulty is that helping that originated through indirect fitness can be later maintained largely by direct fitness^9,22,23^. Another complication is that detecting the preferential helping of relatives (kin-biased helping) is far easier than detecting long-term reciprocal helping over the lifespan, which requires many more observations of helping^24^.

**Extended Data Fig. 1:**
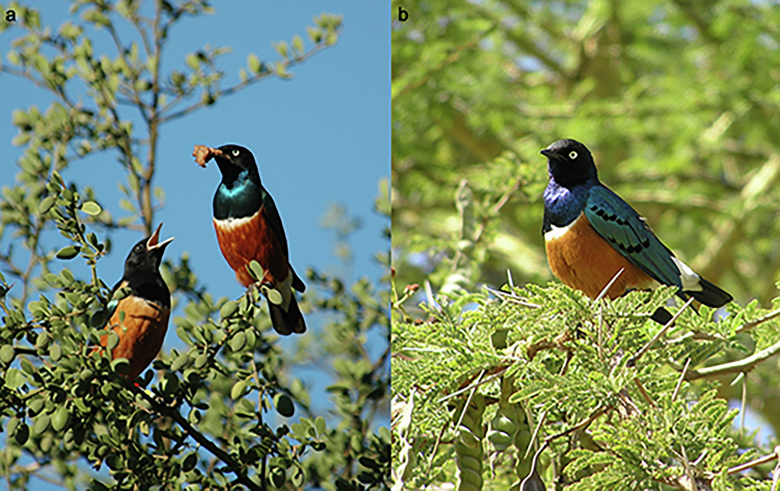
Superb starlings are obligate cooperative breeders, with every breeding pair assisted by up to 16 nonbreeding helpers. **a-b**, Helpers play a critical role in offspring provisioning (of both hatchlings inside the nest and, as the photograph shows, fledglings for a few weeks after leaving the nest) (**a**) and nest defense from predators, where they act as sentinels to guard young from a variety of aerial and terrestrial predators (**b**). Photographs by K. Tseng (**a**) and D.R. Rubenstein (**b**).

Here, we address decades of debate about the roles of direct and indirect fitness benefits of helping behaviour in cooperatively breeding societies^25^ through a 20-year study of the plural breeding superb starling (*L.* *superbus*), a species that forms large, mixed-kin groups with 7 to 60 members (mean group size ranged from 13 to 41 individuals across 9 groups during our study period^26^) including up to 7 breeding pairs per group^26–28^. As superb starlings at our study site in central Kenya breed twice a year during the long and short rainy seasons, we were able to study relationships between breeders and helpers across 40 consecutive breeding seasons. Birds in the population were marked with a numbered metal ring and a unique combination of coloured leg bands, and observed at active nests during the breeding season. Breeding pairs of this obligate cooperative breeder are aided by up to 16 non-breeding helpers (mean number of helpers per nest ± s.e.m. = 4.95 ± 0.21) in both offspring provisioning and nest defence from predators (helping; Extended Data Fig. 1). Helpers provide fitness benefits to breeders by increasing the breeder’s reproductive success and decreasing the costs of reproduction (that is, load lightening), especially for mothers^28,29^. Helping behaviour could also yield direct fitness benefits by increasing group size (group augmentation), as superb starlings in larger social groups have a higher rate of adult survival^30^ and higher reproductive success^31^ than those in smaller groups.

What remains unexplained, however, is variation in helping within groups. Although helpers often aid non-kin, kin discrimination seems likely because past work in this species showed that individuals regularly help their parents and other close relatives^32^, despite low average relatedness (*r*) within social groups (mean pairwise *r* ± s.d. = 0.08 ± 0.04)^33^. We therefore explicitly tested for kin-biased helping within groups. Furthermore, if individuals also preferentially and repeatedly help specific non-kin in their group, then this could indicate the existence of social relationships that provide direct benefits beyond increasing group size. To better understand the mechanisms that underlie cooperation in an avian cooperative breeder, we examined the possible roles of kin selection (kin-biased helping) and reciprocity (reciprocal helping) in the evolution of helping behaviour—the defining characteristic of cooperatively breeding societies.

## Not all helping is kin-biased

To evaluate evidence for kin discrimination in helping behaviour, we fitted a Bayesian negative binomial multilevel model (see Methods for model-fitting details). To predict helping rates while controlling for sampling effort, the response variable was minutes of help given to observed nests each day by each possible helper (that is, those present in the group in a particular breeding season), with the log-transformed minutes of nest observation time each day (exposure) as the offset term. We set the identity of each nest, helper and helper–breeder pair as random intercepts. The fixed effect was kinship between the helper and the parent with the highest degree of relatedness to that helper (scaled and estimated from molecular markers^34^ and pedigrees; see Methods). To ensure that any effect of kinship is not caused by group membership, we scored absence of helping as zero only for non-helping group members that were present that breeding season (see Methods). We used equal-tailed quantiles (2.5% to 97.5%) to estimate Bayesian 95% credible intervals around the mean of the posterior probability distribution.

**Fig. 1:**
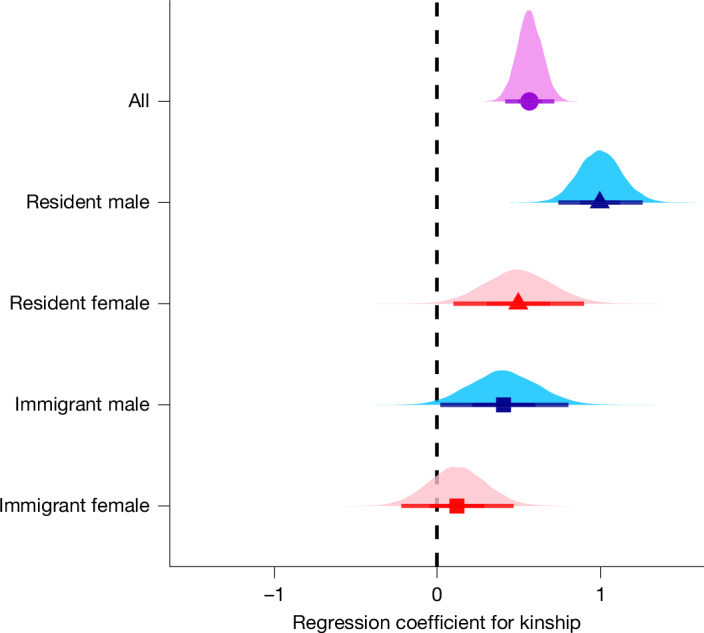
Kin-biased helping in superb starlings. Posterior probability distributions (with means and 95% Bayesian credible intervals) are shown for the standardized regression coefficients for kinship as a predictor of helping, across all helpers (circle, violet), or within residents (triangles) or immigrants (squares) that are male (blue) or female (red). The category ‘All’ includes individuals of unknown dispersal history. The regression coefficient for kinship represents the increase in the helping rate corresponding to a 1 s.d. increase in kinship (for example, from *r* = 0 to *r* = 0.19). Regression coefficients, sample sizes and convergence diagnostics are provided in Extended Data Table 2.

As expected for a cooperative breeder with social groups composed of both kin and non-kin^6^, we found that helpers demonstrated kin discrimination by selectively aiding breeders who were their closer relatives rather than helping group mates indiscriminately (Fig. 1). An increase of 1 s.d. in kinship (for example, from *r* = 0 to *r* = 0.19) between helpers and breeders was associated with a 76% increase in the helping rate (that is, incidence rate ratio (IRR) = 1.761, regression coefficient for kinship = 0.566, 95% credible interval = [0.419, 0.716]). This result indicates a clear role for kin selection in the evolution of helping behaviour in superb starlings.

**Extended Data Fig. 2:**
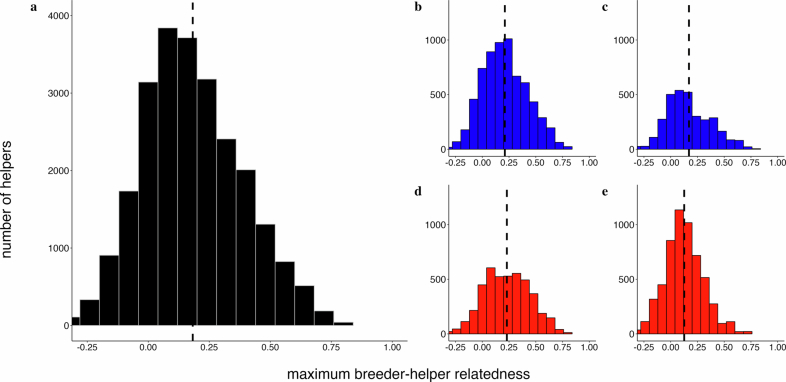
Superb starlings help both kin and nonkin regardless of sex or dispersal history. **a-e**, The distribution of marker-based relatedness estimates in 5889 pairs of breeders and helpers (**a**), 1572 pairs of a breeder and resident male helper (**b**), 819 pairs of a breeder and immigrant male helper (**c**), 1022 pairs of a breeder and resident female helper (**d**), and 1435 pairs of a breeder and immigrant female helper (**e**). Relatedness estimated from 15 polymorphic microsatellite markers, and maximum breeder-helper relatedness is the higher of the two relatedness estimates for either of the two breeding parents to a given helper. The vertical dashed line indicates the mean of the maximum breeder-helper relatedness values.

As low and variable kinship within superb starling social groups results from high immigration of both sexes^26^, we also fitted the same model separately for resident helpers (that is, those born in the group) and immigrant helpers of each sex to determine whether kin-biased helping differed by dispersal history and sex. We predicted that kin-biased helping would be more evident in resident helpers than immigrant helpers because residents have more consistent access to relatives throughout their lives^32^. As predicted, we found clear kin-biased helping in resident helpers of both sexes, although we also found some evidence for kin-biased helping in immigrant males (Fig. 1). Despite evidence of kin discrimination, helpers frequently assisted at the nests of non-kin regardless of sex or dispersal history. For example, 55% of observed helping by resident females and 62% of observed helping by resident males was directed to non-kin (Extended Data Table 1). Overall, a helper’s relatedness to the breeders was highly variable (Extended Data Fig. 2), and the helper was unrelated to both parents in most observations (Extended Data Table 1).

To further evaluate the role of kin discrimination in helping behaviour given the high frequency of helping directed to non-kin, we next examined whether helpers spent more time helping at the nests of kin than at the nests of non-kin on days when both options were available simultaneously. As before, we defined helper–breeder kinship using the relatedness of the helper to their closest relative of the two parents. As categorizing kin and non-kin can use different threshold levels of relatedness, we conducted a sensitivity analysis by repeating the analysis across ten thresholds for defining kinship, ranging from *r* = 0.05 to *r* = 0.5. For each kinship threshold, we calculated means and bootstrapped 95% confidence intervals for the paired difference in helping rates at the nests of kin (above the threshold) and non-kin (below the threshold). Although this approach more directly examined biases in the choices of individuals to help either kin or non-kin, helpers were rarely observed with the opportunity to help at nests of both kin and non-kin on the same day, and this analysis therefore relied on far fewer observations (*n* = 48 to 72 helpers, depending on the kinship threshold).

**Fig. 2:**
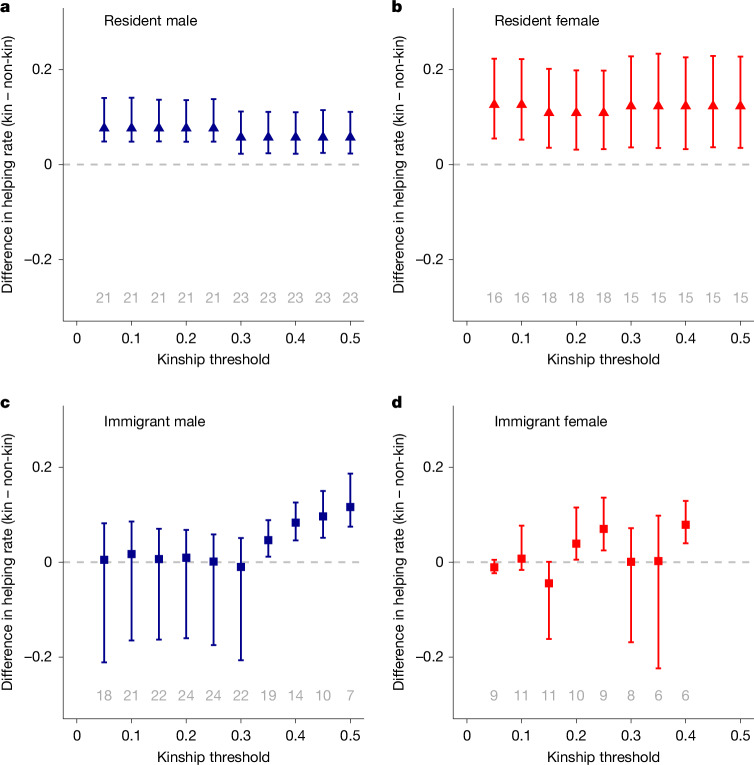
Helpers invested more time helping at kin nests than at non-kin nests when both options were available. Points and error bars show mean and bootstrapped 95% confidence intervals for kinship bias in mean helping rates (that is, the difference in the proportion of time spent at nests of kin versus non-kin when both kinds of nest were present). **a**–**d**, Estimates of kinship bias for varying thresholds for defining kin (a kinship threshold of 0.1 means that relatedness above 0.1 is kin and relatedness below 0.1 is non-kin) for resident males (**a**), resident females (**b**), immigrant males (**c**) and immigrant females (**d**). The grey numbers above the *x* axes show the numbers of helpers of each type observed with opportunities to help both kin and non-kin on the same day. Means and confidence intervals were not calculated in cases with fewer than five helpers (which occurred only for immigrant females with thresholds of *r* > 0.4).

**Extended Data Fig. 3:**
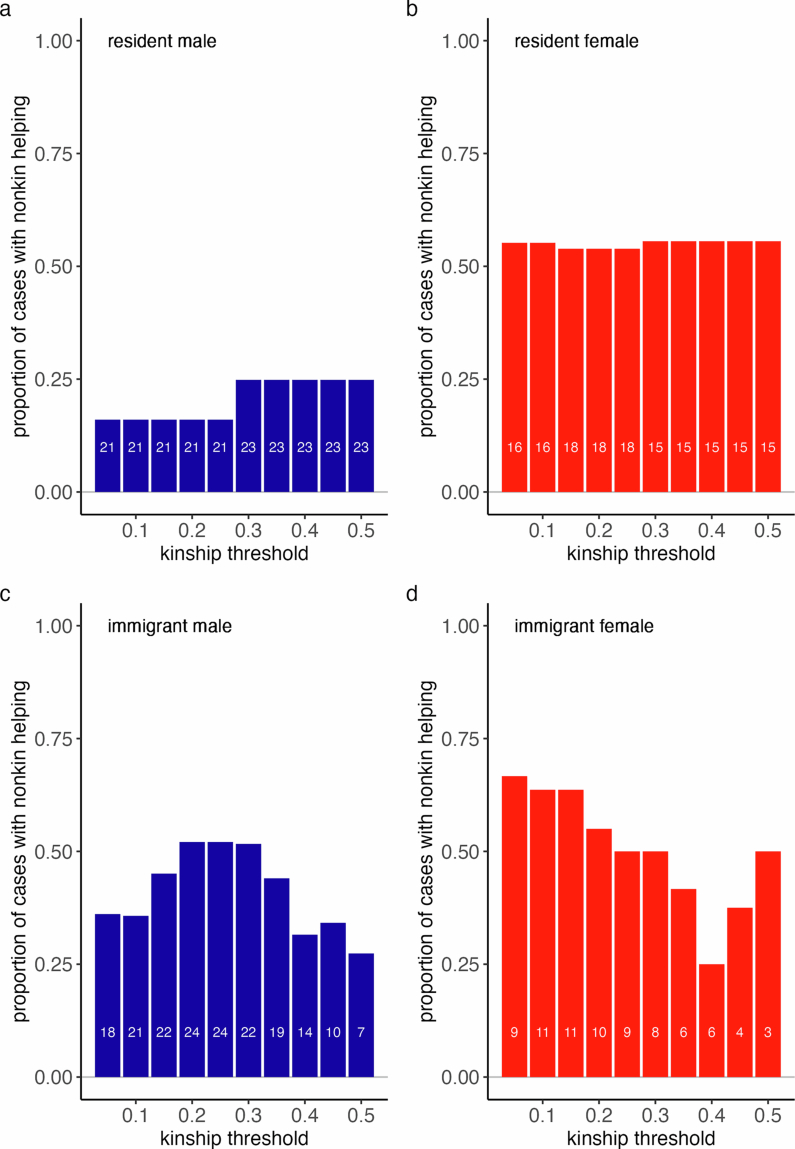
Helpers were more likely to help at kin nests than at nonkin nests when both options were available. **a-d**, Bars show proportion of cases with nonkin helping when kin nests were also present for resident males (**a**), resident females (**b**), immigrant males (**c**), and immigrant females (**d**). Estimates of kinship bias are shown for varying thresholds for defining ‘kin’ (a kinship threshold of 0.1 means relatedness above 0.1 is ‘kin’ and relatedness below 0.1 is ‘nonkin’). The unweighted average probability of nonkin helping across the four helper types ranged from 44% with a kinship threshold of 0.05% to 40% with a kinship threshold of 0.5.

Using this analysis, we again found that resident helpers of both sexes preferred to help kin, and that immigrant male helpers preferred to help particularly close kin (*r* > 0.3; Fig. 2). However, helpers still regularly assisted non-kin, even with simultaneous opportunities to assist kin. Across all threshold-based definitions of kin and across all types of helper, the probability of helping at non-kin nests in the presence of kin nests was high, ranging from 23% to 79% (mean across helper types = 42%, mean across all individuals = 39%; Extended Data Fig. 3). Collectively, the frequency of non-kin helping indicates that helping behaviour in superb starlings cannot be completely explained by kin selection.

## Preference to help specific non-kin

To understand why helpers assisted non-kin even when they had simultaneous opportunities to help kin at other nests, we explored possible direct fitness benefits of helping specific breeders. Past work has shown that individuals in larger superb starling social groups have greater adult survival^30^ and reproductive success^26^. These findings suggest that individuals could augment the size of their social group by helping unrelated breeders (group augmentation), thereby increasing both the helper’s own survival and the survival and reproductive success of their related group mates, and incentivizing immigration from outside groups^23,35^. Given evidence that superb starlings of both sexes can vocally recognize specific individuals^36^, we tested whether helpers had non-random preferences for helping particular unrelated breeders in their groups. To do so, we compared the observed variation in helping across pairs to the distribution of values expected from random helping behaviour within groups. As a null model, we randomized non-kin helping 5,000 times among possible non-kin helpers within each group, nest and day. We found clear evidence for preferred non-kin helping relationships within groups (observed coefficient of variation for helping across dyads = 4.22, *P* < 0.0002, 95% quantiles for expected values = 2.88 to 3.17). Thus, although group augmentation predicts that helping should be based on group membership, it does not explain this relationship-level variation in helping within superb starling social groups.

## Role switching across breeding seasons

**Fig. 3:**
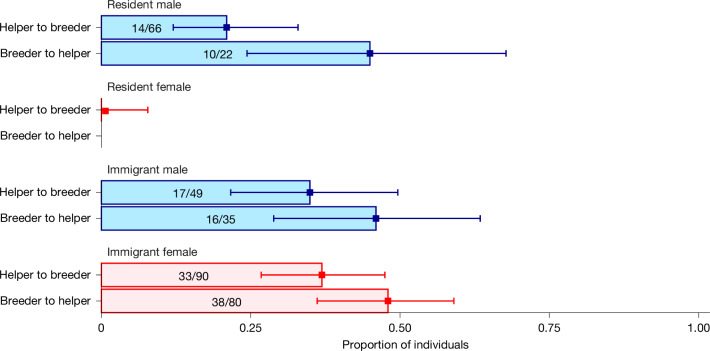
Many individuals of both sexes switched between breeder and helper roles at least once in their lifetime. Bars (blue for males and red for females) show the proportion of individuals of each type that switched between helper and breeder roles across seasons at least once in their lifetime. For simplicity, switches to and from the role of non-breeder–non-helper are not shown. Error bars are 95% confidence intervals from an exact binomial test. To estimate switching probability for lifetimes, only individuals with records for complete lifetimes were included in this analysis.

Given that superb starlings are biased towards helping related group members and towards specific non-kin group members, we next asked whether they might also be biased towards helping group members that are likely to help them in return, irrespective of relatedness. In cooperative breeders, it is generally assumed that helping at the nest cannot be reciprocated between adults because the transitions from helper to breeder roles are typically unidirectional, with subordinate non-breeders initially helping before potentially becoming breeders as they age^37^. In superb starlings, however, breeders occasionally help at the nests of other breeders within a season^32^, and unexpectedly, we found that individual superb starlings frequently switch between breeder and helper roles bidirectionally across breeding seasons. This role switching occurred among individuals of both sexes and both dispersal histories throughout their lifetimes (which can last at least up to 19 years^32^). Most individuals switched roles more than once in their lives (241 of the 329 (73%) individuals for whom we have complete life history data; mean role switches per individual lifetime ± s.e.m. = 3.23 ± 0.17, range = 0 to 18). Notably, at least 44% of males and immigrant females became helpers again at least once after obtaining breeder status (Fig. 3).

Although such role switching across breeding seasons is rarely observed in cooperatively breeding birds^38^, failed breeders in some facultative cooperatively breeding species later help at the nests of their close relatives in the same breeding season (redirected helping)^39–42^. In superb starlings, however, role switching cannot be explained by redirected helping because switches occur most often across breeding seasons, rather than within a season. We observed 249 cases of failed breeding attempts in which individuals were observed again in a later season. Breeders were breeders again as their next role in 97 cases (40%), breeders next became non-breeder–non-helpers (that is, neither bred nor helped) in 91 cases (36%), and in only 61 cases (24%) did they become helpers next. These transition patterns were similar after successful breeding attempts: of the 224 successful breeding attempts in which individuals were observed again in a later season, breeders were breeders again as their next role in 94 cases (42%), switched to non-breeder–non-helpers in 67 cases (30%) and switched to be helpers in 63 cases (28%). Given the low physiological costs of breeding relative to those of other co-occurring starling species that are less social^27,43^, it is also unlikely that transitions from breeder to helper are entirely explained by breeder exhaustion. The breeder-to-helper transitions were instead predicted by an interaction between sex and dispersal history (Fig. 3). Both resident and immigrant males often swapped breeder and helper roles at least once, whereas resident females were almost always helpers and never became breeders (0 of 152 opportunities, 91 individuals, 45 individuals with lifetime data), and immigrant female breeders were just as likely to remain a breeder across seasons (39 of 80 individuals with complete lifetime data) as to become a helper (38 of 80 individuals; Fig. 3).

## Reciprocal helping across years

As frequent role switching creates the potential for reciprocal helping, we looked specifically for cases in which an individual helped a breeder and that breeder later became a helper for that same individual. Of the 528 helper–breeder pairs for which observing reciprocal helping was possible (Methods), we observed reciprocal helping in 142 pairs (19 pairs of females, 50 pairs of males and 73 mixed-sex pairs), including between 2 residents, 2 immigrants or 1 immigrant and 1 resident. As roughly half of the pairs that we observed reciprocating help were same sex, reciprocal helping cannot be completely explained by reproductive motives (for example, helpers promoting future mating with current breeders). The mean observed latency between observations of help given and received was 272 days, about two breeding seasons.

To test whether reciprocal helping predicted greater helping rates within and across different types of helper (that is, resident and immigrant males and females), we fitted a similar model to the one described above for kin-biased helping (response was daily helping minutes; offset was daily observation minutes; random intercepts were helper, nest and helper–breeder pair), but we replaced the fixed effect of kinship with reciprocal help as a binary variable indicating the presence or absence of reciprocal help from either parent at any time during the study. We did not define reciprocal help using estimates of exact observed durations of help received in a previous season for two reasons. First, we did not expect that reciprocal helping would be based on individuals remembering and precisely matching their daily helping rates with others across seasons. Second, using the presence or absence of a reciprocal helping relationship reduces residual confounding because we lacked the sampling necessary to accurately estimate exact reciprocal helping rates within each helper–breeder pair over the entire nest attempt. As in our kin-biased helping model, we coded non-helping as zero only for group members that were present to help, such that any evidence of reciprocal helping could not be caused by group structure (Methods).

Using this analysis, we found that specific pairs of individuals maintained long-term reciprocal helping relationships by swapping helper and breeder roles across their lifetimes. All else in the model held constant, the rate of helping was 242% greater for reciprocating pairs than for pairs that did not reciprocate (IRR = 3.42, regression coefficient for reciprocal help = 1.23 [0.76, 1.70]).

**Fig. 4:**
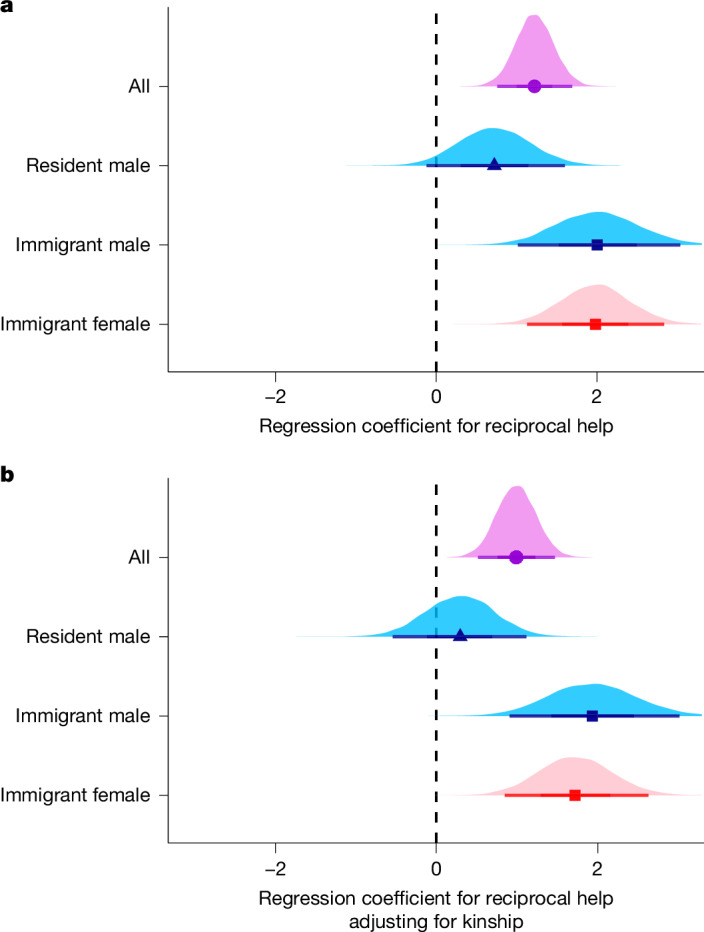
Reciprocal helping is not driven by kinship. **a**, Posterior probability distributions (with means and 95% Bayesian credible intervals) for the regression coefficients for reciprocal help as the only predictor of helping. These coefficients represent the increase in the helping rates to nests in which one of the recipient parents was a reciprocal helper in the past or future. Resident females could not reciprocate because they were never breeders. **b**, Posterior probability distributions (with means and 95% Bayesian credible intervals) for the regression coefficients for reciprocal help as a predictor of helping when adjusting (statistically controlling) for kinship. The similarity in estimates between the top and bottom panel shows that reciprocal help is not driven by kinship. Estimates are shown across all helpers (circle, violet), including individuals of unknown dispersal history, as well as for male (blue) and female (red) helpers who are residents (triangles) or immigrants (square). The same figure with coefficients of kinship is shown in Extended Data Fig. 6. Regression coefficients, sample sizes and convergence diagnostics are provided in Extended Data Table 2.

**Extended Data Fig. 4:**
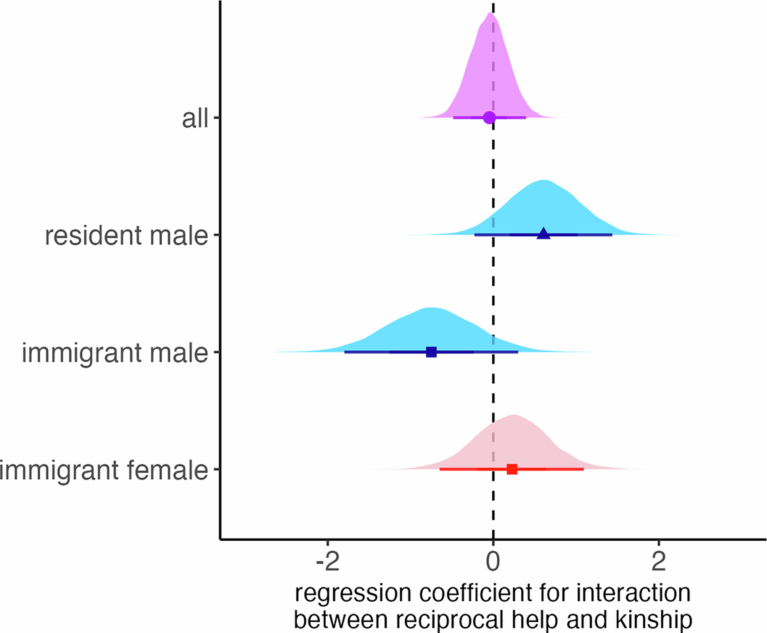
No clear evidence that kinship impacts the probability of reciprocal helping. Posterior probability distributions (with means and 95% Bayesian credible intervals) are shown for a possible interaction between kinship and reciprocal help across all helpers (circle, violet), including individuals of unknown dispersal history, as well as for male (blue) and female (red) helpers who are residents (triangles) or immigrants (square). A positive or negative estimate would indicate that greater kinship increases or decreases the probability of reciprocal helping versus one-way helping. Regression coefficients, sample sizes, and convergence diagnostics are provided in Extended Data Table 2.

**Extended Data Fig. 5:**
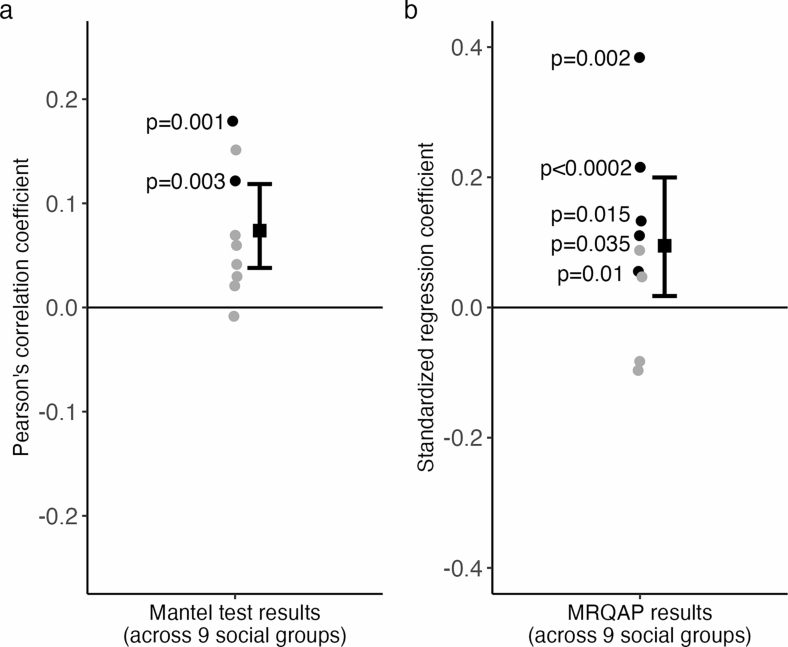
Nonparametric network permutation tests within nine social groups confirm reciprocal helping. **a**, Results of Mantel Tests applied to each of nine social groups. Points show Pearson’s correlations between given and received help. **b**, Results of multiple regression quadratic assignment procedure (MRQAP) with double semi-partialling applied to each of nine social groups. Points show standardized regression coefficients for effect of help given on help received after controlling for effect of kinship. In both plots, dark points with p-values show groups where test was statistically significant, and squares show the mean for the observed statistic across the nine groups with bootstrapped 95% confidence intervals.

As reciprocal helping can, and did, occur between relatives, we next separated the roles of reciprocal help and kinship as predictors of superb starling helping behaviour by testing for evidence of reciprocity while statistically controlling for kinship. When we fitted the same models including both reciprocal help and kinship as predictors, we found that helping was still clearly reciprocal (Fig. 4b), and that the helping rate was 169% greater for reciprocating pairs than for pairs that did not reciprocate (all individuals: IRR = 2.69, coefficient for reciprocal help = 0.99 [0.52, 1.47]; see Methods for interpretation of regression coefficients). Although reciprocal helping was evident overall across helpers (Fig. 4a), this pattern was driven by immigrants. By contrast, the effect of reciprocal help was confounded with kinship in resident males (Fig. 4b), and reciprocal helping was absent in resident females because females never became breeders in their natal groups (Fig. 3). We did not detect evidence for an interaction between reciprocal help and kinship in resident males and immigrants (Extended Data Fig. 4; coefficient for interaction = −0.05 [−0.50, 0.39]), meaning that reciprocal helping occurred among both kin and non-kin. To test the robustness of our finding that reciprocal helping was not explained by kinship, group structure (geographic distance) or biased opportunities to be seen helping, we also reanalysed our data using a more conservative non-parametric permutation test that was developed to account for sampling biases. This null hypothesis test confirmed the conclusions of our statistical model (see Methods and Extended Data Fig. 5 for results). To our knowledge, this form of reciprocity has never before been discovered in a cooperatively breeding society.

**Fig. 5:**
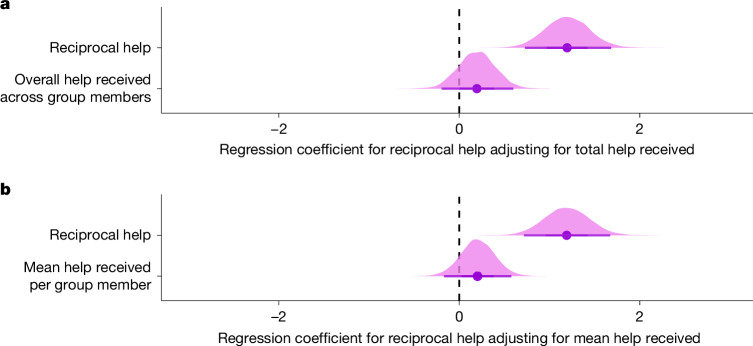
Reciprocal helping is not explained by generalized reciprocity at the group level. **a**,**b**, Posterior probability distributions (with means and 95% Bayesian credible intervals) for the regression coefficients for reciprocal help and either the sum of helping rates (seconds of helping/seconds of observation) across group members (**a**) or the mean of helping rates (seconds of helping/seconds of observation) across group members that could have helped (**b**). Regression coefficients, sample sizes and convergence diagnostics are provided in Extended Data Table 2.

Under some scenarios, natural selection acting in group-structured populations can favour individuals that help others on the basis of the amount of help that they receive from any or all group members (generalized reciprocity)^44^, which could drive patterns of reciprocal helping in a cooperative breeder. To assess this alternative mechanism, we fitted two models identical to our reciprocal help model described above, but with either the total or mean help received added as a covariate. The first model included reciprocal help and the sum of helping rates to that helper. The second model controlled for the helper’s group size by including reciprocal help and the mean of helping rates to that helper averaged across possible social partners. In both models, reciprocal help from specific group members was a stronger predictor than the total help received from group mates (model 1: coefficient for reciprocal help = 1.20 [0.71, 1.69]; coefficient for standardized total help received = 0.20 [−0.2, 0.59]; model 2: coefficient for reciprocal help = 1.19 [0.73, 1.67]; coefficient for standardized mean help received = 0.20 [−0.15, 0.57]; Fig. 5). Thus, we found that reciprocal helping in superb starlings could not be explained by generalized reciprocity at the group level.

## Direct versus indirect fitness

Together, our results indicate that the relative importance of direct versus indirect fitness benefits varies with a helper’s sex and dispersal history. Resident females preferentially helped kin and never engaged in reciprocal helping because they never became breeders. Resident males also preferentially helped kin and sometimes swapped breeder and helper roles with both kin and non-kin, but not often enough for reciprocal helping to be a clear predictor of helping rates. By contrast, immigrants showed clear evidence of reciprocal helping with both kin and non-kin, and only limited evidence of kin-biased helping. Immigrant females reciprocated help, but they did not show clear kin-biased helping across all days and nests, and we found only weak evidence for kin-biased helping on days when they had opportunities to help both kin and non-kin simultaneously (Extended Data Fig. 2). Immigrant males demonstrated both reciprocal helping and kin-biased helping (Fig. 1 and Extended Data Fig. 2). Kin-biased helping in immigrant males is unlikely to be explained by helping their adult resident offspring: of the 47 pairs in which an immigrant male engaged in reciprocal helping with another immigrant, only 15 were closely related (*r* > 0.125). Instead, this pattern of kin-biased helping in immigrant males (Figs. 1 and 2) suggests that some individuals either recognized kin that joined the same group at another time or dispersed with kin (referred to as budding dispersal^45^ or dispersal coalitions^46^). Although dispersal coalitions in superb starlings have not been explored in males, previous work on females showed that sisters disperse together in coalitions or immigrate into the same groups in successive years^47^, probably recognizing individuals—rather than just kin—through their flight calls^36^.

Helpers of both sexes and dispersal histories, including resident females who did not engage in reciprocal helping, often aided non-kin breeders even when they simultaneously had the option to help kin. This pattern suggests a role for other sources of direct fitness benefits beyond reciprocal helping, such as group augmentation. Indeed, past work in superb starlings suggests that direct fitness benefits of group augmentation are critical for the evolution of their mixed-kin societies^26^, and therefore might also promote helping behaviour and cooperation more generally. The ability of immigrants to form reciprocal helping relationships could be an additional benefit of joining or maintaining larger groups. Immigrants are crucial for group stability because offspring recruitment alone is insufficient to prevent group extinction^26^, and help directed from residents to immigrants could encourage them to stay in the group. Previous studies in superb starlings also show that male helpers can occasionally gain direct fitness benefits in the form of extrapair paternity by investing their helping effort in unrelated female breeders^33,48^. Furthermore, there may be additional sources of direct fitness benefits for helpers that have yet to be explored in superb starling societies, such as signalling of helping ability. Ultimately, given that past work in this population highlights the potential for extrapair mating by helpers^48^ and the importance of group size^26^, and given that the current study demonstrates frequent helping directed to non-kin and the existence of reciprocal helping among both non-kin and kin, we suggest that helping behaviour in superb starlings is more influenced by direct fitness benefits than by indirect fitness (kin selection).

## Reciprocal helping in other species

Although non-kin helping has been reported in other avian cooperative breeders, evidence of reciprocity as the underlying mechanism is lacking. For example, in the pied kingfisher (*Ceryle rudis*), helping non-kin is thought to improve future breeding chances but not reciprocal help^49^. Within-season reciprocal helping occurs in both white-fronted bee-eaters (*Merops bullockoides*)^41^ and bell miners (*Manorina melanophrys*)^50^, but for both species, kin selection has been shown to be more important than any potential role of reciprocity, for which evidence was weak. In the green woodhoopoe (*Phoeniculus purpureus*), helpers may compete for opportunities to feed nestlings because doing so increases the chance that, when these nestlings mature, they will disperse with and later help raise the offspring of the helper who assisted in raising them^20,51^. However, the role for reciprocity in driving these helping relationships has subsequently been questioned^52^. Like superb starlings, all of these species are obligate cooperative breeders living in mixed-kin societies in harsh and variable environments in which helpers are necessary for successful reproduction. Forming reciprocal helping relationships with non-kin may therefore ensure that the necessary help is available in frequent but unpredictable times of need, as it does in superb starlings^26^.

## Reciprocity or pseudo-reciprocity

The reciprocal helping relationships observed in superb starlings are consistent with help given to a recipient causing help to be received from that recipient (that is, reciprocity). However, an alternative and often-favoured explanation for this and all other putative cases of reciprocity, in cooperative breeders and elsewhere, has been pseudo-reciprocity (that is, when help given to a recipient increases by-product benefits from that recipient)^11^. Pseudo-reciprocity is not only likely to be common in natural systems, it is also a difficult general explanation to reject because there are many possible by-product return benefits that can be reasoned to result from helping, including access to future breeding positions, mates or resources^37^. However, both theory and empirical evidence suggest that the existence of by-product benefits (including those from group augmentation) does not negate the additional benefits of conditionally investing in, choosing or switching partners on the basis of past helping behaviour^1,13–16^. When animals form long-term cooperative relationships, helping can both enable some by-product benefits (pseudo-reciprocity) and promote some reciprocal help (reciprocity)^13–16^. In such cases, the by-product benefits of each partner’s existence can cause helpers to have some amount of stake in their partner’s health and survival (fitness interdependence) that is independent of the partner’s behaviour^53^, and also show some amount of behavioural responsiveness to the partner’s actions (for example, by gradually switching from less helpful to more helpful partners^13–16,54^). As increasing evidence supports the view that indirect fitness, by-product benefits and reciprocal investments can additively or synergistically stabilize many cooperative traits, the relevant question concerns not which factor explains helping but rather the relative roles of each factor^13–16^.

Our findings also raise the question of whether the relative ease of detecting evidence for kin-biased helping and group augmentation has led reciprocity to be prematurely rejected as an additional potential factor promoting helping in cooperative breeders. Many authors have long considered reciprocity to be too cognitively demanding for non-human animals, but this assumption applies only to so-called calculated reciprocity^55,56^. Controlled studies across a range of species show that many non-human animals prefer to associate with or help partners that reciprocate help over partners that do not^55^. These biases towards more helpful partners can arise from simple, widespread and evolutionarily conserved cognitive mechanisms such as associative learning (for example, avoiding partners associated with bad experiences)^16,55^. Moreover, studies of interspecific mutualisms show that even organisms without cognition have repeatedly and independently evolved the ability to enforce cooperation through conditional partner choice^1,55^.

A second misunderstanding that makes reciprocity unnecessarily controversial is that most debates about reciprocity still view it as a standalone alternative to kin selection or by-product benefits, such that any evidence for these other explanations is counted as evidence against any causal effect of help given on help received^15,55^. This view of reciprocal help, indirect fitness and by-product returns as alternatives rather than complementary mechanisms clearly affected some of the early studies that addressed these ideas in avian cooperative breeders^41,50^. Yet, the evolution of reciprocity requires the existence of one-way helping behaviours that must have initially evolved either through kin selection or by-product benefits^9^, and so for cases in which reciprocity exists, the coexistence of kin selection or by-product benefits should be the expected norm. There are several ways that either kin selection or by-product benefits (for example, group augmentation) could pave the way for the evolution of reciprocity in superb starlings or other cooperative breeders^9,22^. One example is the kinship deceit hypothesis^57^ that posits that kin discrimination might be based on associations learned at the nest, and if so, that helping at the nest can benefit unrelated helpers when it triggers kin discrimination heuristics that cause future reciprocal helping from the parents or the nestlings^51,58^. Although this scenario has been referred to as deceit, it is also reciprocity in the broad sense that the help given to the recipient causes the help received from the recipient. Similarly, group augmentation in superb starlings^26^ could lead to reciprocity if helping decisions are biased by cues of group membership and receiving help becomes one such cue. As a consequence, individuals would be more likely to help partners that help them.

Separating the precise mechanisms that shape reciprocal helping decisions in superb starlings or other cooperative breeders will require future experimental manipulations of natural helping behaviour. Given the role of acoustic signals and vocal recognition in superb starlings^36^ and other avian cooperative breeders^59,60^, playbacks could be used to measure responsiveness to manipulated helping or simulate the presence of birds that are not actually able to help. For example, in the cooperatively breeding dwarf mongoose (*Helogale parvula*), researchers used playbacks to experimentally increase acoustic cues of vigilance behaviour in specific individuals, which caused them to receive more social grooming from their group mates later that day^21^. A question for future research is whether conditional shifts in cooperative behaviour also occur over the longer time periods documented here.

To summarize the debate about reciprocity, the game theoretic strategy tit for tat in the repeated prisoner’s dilemma^9^ led many empiricists to consider only a narrow, literal and extreme model of reciprocity in which strictly conditional and binary responses to defection occur in a high-conflict scenario with no partner choice^55^. Under more nuanced and ecologically realistic conditions, however, individuals can form long-term social relationships that involve some responsiveness to partner defection, some stake in the survival of certain partners (that is, fitness interdependence)^53^ and some ability to choose or switch among partners, leading to market forces^54^. In these cases, reciprocity (as responsiveness to partner help) is one causal effect in a larger model. More recent models that integrate the evolution of helping behaviour and the formation of preferred partnerships (social bonds) allow reciprocity to evolve and interact with other factors^16^, showing that long-term reciprocal helping relationships (with intermediate levels of partner responsiveness) can change over evolutionary time as cooperative traits evolve and also over developmental time as individuals learn and adjust their behaviour across partners^14,15^. As unconditional helping foregoes the potential benefits of having a more cooperative partner, a helper can still benefit from gradually switching from less helpful to more helpful partners, even when establishing new partnerships takes a substantial amount of time^13–16^. Such gradual partner switching is a form of reciprocity that is hard to measure from field observations alone.

## Detecting reciprocal helping

**Fig. 6:**
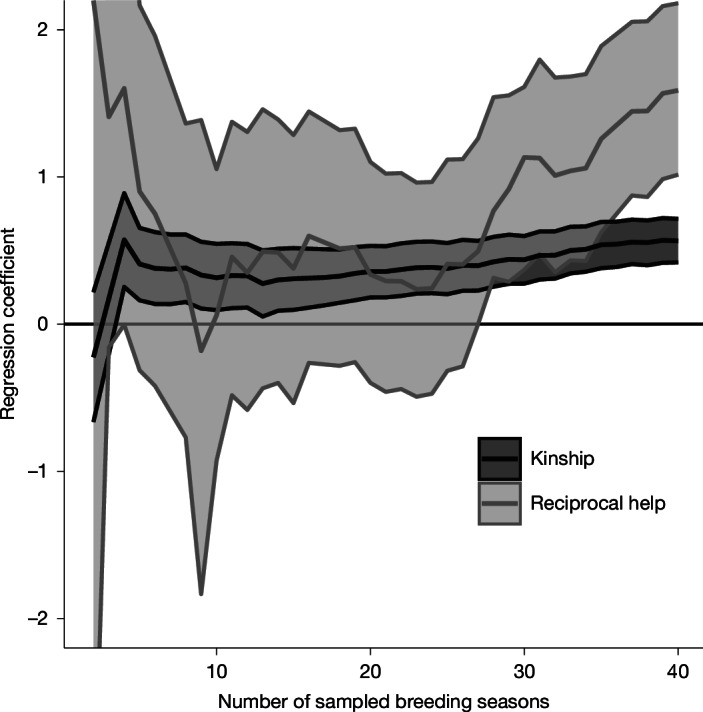
Power analysis based on resampling. Far fewer observations are needed to detect kin-biased helping than to detect reciprocal helping, despite kinship having a smaller effect size. Means and 95% credible intervals for the effect of kinship (black) and the effect of reciprocal help (grey) on helping rates estimated for subsamples of the total dataset increasing in size from 1 to 40 breeding seasons.

In observational studies, kin-biased helping can mask evidence for reciprocity because kinship and help received are confounded when reciprocal helping occurs between relatives, and because detecting kin-biased helping requires far fewer observations of help than does detecting reciprocal helping^24^. As a consequence, short-term studies can easily discover kin-biased helping but fail to find evidence for reciprocal helping, obscuring the roles of direct and indirect fitness^24^. In superb starlings, the timescales of reciprocation occurred over years. Therefore, we detected reciprocal helping only because of our long-term field study: >12,000 helping observations across 563 helpers at 410 nests, across 9 social groups over 40 breeding seasons spanning 20 years. Without such an extensive record, reciprocal helping over such long and variable time spans would be nearly impossible to detect, and any reciprocal helping between relatives would simply be explained as kin-biased helping. In our study, kin-biased helping was evident after only 3 breeding seasons, but detecting evidence for reciprocal helping required at least 27 breeding seasons (Fig. 6). Moreover, the effect of kin-biased helping was relatively stable after 5 breeding seasons of data collection, but the effect of reciprocal helping was still trending upwards after 40 breeding seasons, suggesting that the role of reciprocal helping was underestimated even after 20 years of continuous data collection. Indeed, even after 10 years (20 breeding seasons) of observation, an analysis of our data would infer clear kin-biased helping, but not reciprocal help (Fig. 6). This finding highlights the importance of long-term field studies.

## Conclusion

Our findings confirm a role for kin selection, but also challenge the common assumption that kin selection (indirect fitness) is sufficient to explain helping behaviour in vertebrate cooperative breeders^25^. The view of helping purely as a form of altruism due to kin selection is contested by our discovery of cryptic long-term reciprocal helping between both related and unrelated group members. Although some theoretical^8^ and empirical^61^ studies focus on direct fitness mechanisms underlying the evolution of cooperatively breeding societies, the potential for reciprocity is rarely considered as an explanation for helping behaviour in cooperative breeders. Using 40 breeding seasons of continuous data on helping behaviour over 20 years in cooperatively breeding superb starlings, we showed that: non-kin helping was common even in the presence of kin and despite a clear capacity for kin discrimination; individuals formed long-term non-random reciprocal helping relationships by swapping breeder and helper roles across breeding seasons and years; patterns of kin-biased helping and reciprocal helping varied with helpers’ sex and dispersal history; and the discovery of reciprocal helping in cooperative breeders was possible only with long-term field observations. Taken together, the findings of our study highlight the need to integrate analyses of multiple mechanisms for both direct and indirect fitness to gain a more nuanced understanding of the evolution of cooperation and of animal societies. Even though cooperative breeders often live in family groups, increasing empirical evidence shows that direct fitness plays a larger role in the evolution of cooperatively breeding societies than previously realized, both in terms of group formation^8,26^ and in helping behaviour^7^. Alongside group augmentation, we suggest that within-group reciprocity may be a cryptic but crucial source of direct fitness that promotes the stability of complex cooperative societies.

## Methods

### Field data collection

From 2001 to 2021, we built a longitudinal dataset of nine social groups of superb starlings at the Mpala Research Centre in Laikipia, Kenya (0° 17′ N, 37° 52′ E)^32^. We used baited pull-string traps (during the non-breeding season) or mist nets (at active nests during the breeding season) to capture individuals first observed in this population after immigration or fledging. We also banded individuals as hatchlings in the nest during the two annual breeding seasons (short rains breeding season: October–December; long rains breeding season: March–June)^62^. Each of the 1,175 individuals in the population was marked with a numbered metal ring and a unique series of coloured leg bands^32^. As a result, we gathered fine-scale, individual-level behavioural observation data from focal observations and daily nest checks during breeding, as well as year-round opportunistic census observations and data collected during trapping. Banding of this population began in 2001 and focal observations began in 2002.

We conducted focal observations of nests during breeding using a spotting scope. Focal observations were typically 120 min in length (median = 120 min, mean = 125 min, range = 3 to 240 min, 90% were at least 120 min, 99.5% were at least 60 min). For each individual observed within 30 m of the nest, we recorded their identity, group membership, times of arrival and departure, their breeding role (that is, helper or breeder) and whether they arrived with food. Over 730 days from 2002 until 2021 (40 breeding seasons), we observed 410 nests in the 9 social groups and analysed 12,112 visits to the nest with complete data (out of 14,588 events). In total, we observed 563 individually banded superb starlings acting as helpers and 254 as breeders over the course of the 20-year study.

Helpers are alloparents observed visiting active nests during breeding to provide offspring provisioning and/or nest guarding^30^. Breeders are the mother (the female that incubated during the egg stage) and father (the male guarding the incubating female during the egg stage). We confirmed observed parentage genetically for all chicks that survived to reach seven days old (see below for details). Within-group extrapair paternity by helpers was low, accounting for fewer than 7% of all offspring, and we found no evidence that more than one female lays eggs in a nest^48^. For each active nest, we inferred the possible helpers present at the nest by first aggregating all observations of individuals observed within groups, and then assuming that individuals were present within a group throughout the breeding season, between their first and last season observed. To be conservative and consistent with previous studies in this system^30,47,63^, we assumed that any individual not seen for five or more seasons was dead, and that any subsequent isolated single observations were misidentifications. We showed previously that results were robust to this assumption, as they did not change after performing a sensitivity analysis in which we extended the length of subsequent breeding seasons required before assuming that an individual had died^30^.

### Determining sex and dispersal history

Sex was verified genetically for all individuals using PCR^64^ as previously described for this species^48^. Dispersal history includes immigrant (banded as juveniles or older, with parents genetically identified as not belonging to the same group), resident (banded as hatchlings in the nest, or banded as juveniles whose parents were genetically identified as members of the same group) and unknown (individuals who already existed in the population at the beginning of the study before dispersal could have been observed). We labelled 21 females who already existed in the population at the beginning of the study and that became breeders as immigrants because we observed that resident females never became breeders (0 of 91 individuals, 0 of 152 cases, binomial test: 95% confidence interval = 0% to 2% chance), whereas 68 of 112 immigrant females became breeders (binomial test: 95% confidence interval = 51% to 70% chance). If the probability of breeding as a resident female is 0% to 2%, then each of these females has a 97.4% to 100% chance of being an immigrant.

### Estimating helping

We defined helping as the minutes of provisioning and/or nest defence during a focal observation period (Extended Data Fig. 1). We rounded observations up to the nearest minute. For instance, if an individual was observed feeding chicks at the nest or bringing food to the nest for less than 30 s, and nest attendance was scored as zero minutes, then we scored help as one minute rather than zero minutes. Observing reciprocal help was possible for a pair of birds when a focal bird was observed helping a partner and in another season, the focal bird was a breeder when the partner was both present and not breeding.

We combined minutes of chick provisioning and nest attendance (nest defence) because estimating dyadic helping rates should seek to maximize the number of potential helping interactions^65–67^, and these behaviours are both clear measures of helping^29^ that we found to be highly correlated. Compared to nest attendance rates, chick provisioning rates are based on fewer data points (less sampling) and are therefore less precise (for example, more likely to falsely represent a low helping rate as a zero). Moreover, across helper–nest pairs, the rate of nest defence explained 51% (95% credible interval = [0.50, 0.53]) of the variation in provisioning rates. To estimate this relationship, we fitted a Bayesian negative binomial multilevel model (using the brms R package^68^ with default priors) with total seconds of provisioning time by a visitor to a nest as the response variable, log-transformed total minutes of sampling time each day as the offset term, scaled minutes of nest attendance as the fixed effect, and nest and helper as random intercepts. To estimate the variance explained by the fixed effect, we then calculated the marginal *R*^2^ and its Bayesian credible interval using the performance R package^69^. We fitted the model using 4 Markov chain Monte Carlo chains with 5,000 iterations and a warm-up of 1,000 iterations, Rhat was 1.00, and the conditional *R*^2^ for the full model was 0.97 (95% credible interval = [0.97, 0.98]). Thus, given the strong correlation between chick provisioning and nest attendance, the much greater sampling of nest attendance per individual and the greater ease of identifying the individuals during nest attendance, we used a combined minutes of chick provisioning and nest attendance to estimate helping behaviour.

### Estimating kinship

We estimated pairwise genetic relatedness in the R package related^52^ from 15 polymorphic microsatellite markers developed for superb starlings using DNA extracted from whole-blood samples (Extended Data Fig. 2); the same markers were also used to confirm parentage^48,70^ in Cervus v3.0.7 (ref. ^71^). Previous work has shown that this panel of superb starling microsatellite markers gives qualitatively similar results to a larger panel of single nucleotide polymorphisms for both relatedness and parentage estimates^72^. We also constructed pedigrees using behavioural observation data confirmed with parentage estimates, and then calculated pairwise kinship using the kinship2 R package^73^. In our models for kin-biased helping and reciprocal helping, we used microsatellite estimates of relatedness for immigrant helpers, and pedigree estimates of kinship for all other birds. Using microsatellite estimates of relatedness for all birds rather than pedigree kinship estimates gave qualitatively similar results and did not affect our conclusions.

### Model-fitting procedure

We fitted all Bayesian multilevel models using the brms R package^68^ with default uninformative priors. We used the R package performance^69^ to compare the fits of quasi-Poisson and negative binomial models for overdispersed count data. Model diagnostics were better for the negative binomial model, and the negative binomial fit predicted the frequency of zero response data better than did a quasi-Poisson model. We fitted all Bayesian models using 4 Markov chain Monte Carlo chains with 5,000 iterations and a warm-up of 1,000 iterations. Across all models in our study, Rhat ranged from 1.000 to 1.001, bulk effective sample size ranged from 4,008 to 20,440, and tail effective sample size ranged from 6,000 to 13,439, indicating model convergence and that estimates of posterior quantiles are reliable.

### Interpreting regression coefficients as effects of kinship and reciprocal help

**Extended Data Fig. 6:**
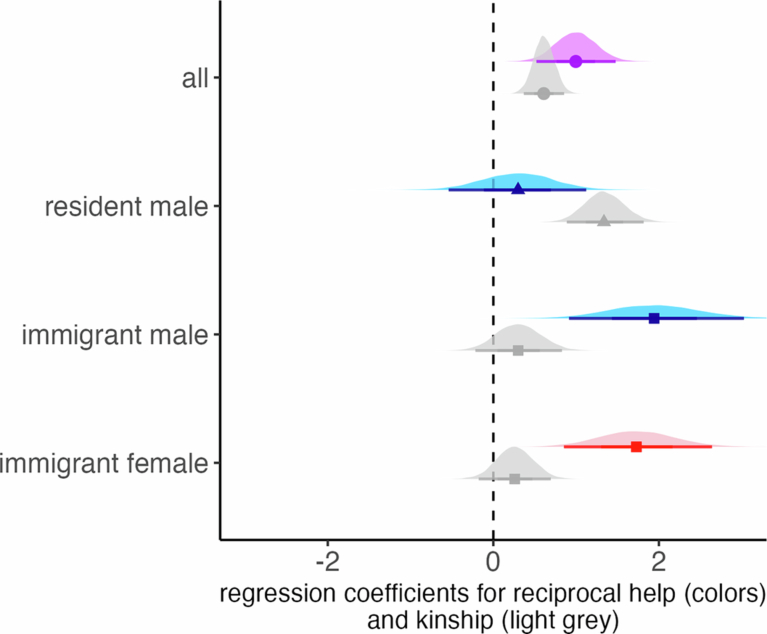
Reciprocal helping is not driven by kinship (shown with regression coefficients for kinship effect). Posterior probability distributions (with means and 95% Bayesian credible intervals) are shown for the regression coefficients for reciprocal help as a *predictor of helping when adjusting (statistically controlling) for kinship*. Estimates are shown for all helpers (circles, violet), including individuals of unknown dispersal history, as well as for male (blue) and female (red) helpers that are residents (triangles) or immigrants (squares). Estimates of the coefficients for kinship as a predictor of helping when adjusting for reciprocal help (grey) should not be interpreted as measures of kin-biased helping, because they exclude pairs where reciprocal help was impossible (e.g., all resident females), and exclude any possible effect of kinship causing help through reciprocal help among kin. Regression coefficients, sample sizes, and convergence diagnostics are provided in Extended Data Table 2.

The regression coefficients from our statistical model with both kinship and reciprocal help must be interpreted carefully with several caveats in mind. The model was designed to assess the effect of the presence of reciprocal help while controlling for kinship. One cannot use the coefficients to directly compare the effect sizes of kinship and reciprocal helping because these effects exist on different scales and are confounded. The regression coefficients in the kinship model (Fig. 1) can overestimate the total effect of kinship on helping because the estimate assumes that all reciprocal helping among kin is caused by kinship (whereas, in reality, some unknown amount of the reciprocal helping between kin could be due to reciprocity). By contrast, the regression coefficients for kinship in the model with both kinship and reciprocal help (shown in Extended Data Fig. 6) underestimate the total effect of kinship, for two reasons. First, this estimate assumes that reciprocal helping among kin is not caused by kinship (whereas, in reality, it might be). Second, estimating the simultaneous effect of reciprocal help in the model requires excluding pairs for which reciprocal help was impossible (that is, all resident females), which is also the subsample of the data in which kinship effects are expected to be largest. As the regression coefficients for kinship in this model cannot be interpreted as the total effect of kinship, we show them in grey in Extended Data Fig. 6.

### Alternative and conservative non-parametric hypothesis-testing procedure

We reanalysed our data using non-parametric network permutation tests designed to remove sampling biases^74^. Observational datasets of social behaviour in the field typically suffer from many sampling biases that can lead to type 1 and 2 error^74^. For instance, the helping rate between two superb starlings depends on how often those two individuals overlapped in space and time and how often an observer was likely to see one individual rather than another. These sources of bias can be removed by including them either in the statistical model (as covariates or random effects) or in a null model (such that they cannot explain the difference between observed and expected statistics). To check the robustness of our overall findings from our parametric statistical model, we used a non-parametric double permutation test^74^ to remove effects of sampling biases (first data permutation) and to remove effects of actor and receiver (second network permutation). To do this, we first calculated an adjusted helping score as the observed helping rate minus the median of 5,000 expected helping rates from a permutation-based null model. The null model swaps helping rates between possible group members within each group, nest and day. In other words, adjusted helping rates indicate how many more helping minutes were observed than what would be expected if nests received the same amount of help from random group members present in the group on each observation day. We then analysed the adjusted helping scores within each group using two non-parametric network permutation tests. The first test was a Mantel test (vegan R package^75^) for the Pearson’s correlation between adjusted helping scores given and received within each group (Extended Data Fig. 5a). The second test was a multiple regression quadratic assignment procedure with double semi-partialling (MRQAP), using the asnipe R package^76^ to test for the effect of adjusted help-given score and kinship (both scaled to remove units) on adjusted help-received score within each group (Extended Data Fig. 5b). We used non-parametric bootstrapping to estimate a 95% confidence interval around the mean across the nine groups. This non-parametric approach is more conservative than our model-fitting approach, and it confirmed the findings from our main statistical model that reciprocal helping was both evident and not driven by kinship or by sampling biases (Fig. 5).

### Statistical power for detecting kin-biased helping and reciprocal help

To demonstrate the difference in power for detecting kin-biased helping and reciprocal helping, we fitted our two Bayesian models (described in the main text) for detecting the effect of kinship or reciprocal help, each 40 times, using increasing subsamples of the data from 1 breeding season to 40 breeding seasons. We then plotted the estimate and 95% credible intervals for each cumulative number of seasons. When visualizing the results for the 80 estimates, we excluded 3 estimates for the effect of kinship and 4 estimates for the effect of reciprocal helping for which there was a lack of convergence when fitting the model.

The large difference in power for detecting kin-biased helping and reciprocal helping (Fig. 6) occurs because reciprocal helping requires more repeated observations per pair^24^. Specifically, the statistical power to detect kin-biased helping is limited by the reliability of kinship estimates, the variation in kinship and the number of individuals and pairs in the dataset, whereas the power to detect reciprocal helping is limited primarily by the number of possible observations of reciprocal helping per pair (that is, helper A helps breeder B and then B can help A). For this study (and most other similar field studies), kin-biased helping should be much easier to detect than reciprocal helping because we have many individuals and many breeder–helper pairs in which helping was possible, but relatively few observations per pair. Most pairs of breeders and helpers were observed together on only 1 day (range = 1 to 18 days, median = 1 day, mean = 2 days). This sparse sampling per pair causes rates of helping and reciprocal helping to be imprecise, and many helping rates estimated at zero were probably not zero. Thus, our estimates of the effect of reciprocal helping are probably more conservative than that of kinship, making it even more unexpected that we detected such strong evidence of reciprocity in this study.

### Reporting summary

Further information on research design is available in the Nature Portfolio Reporting Summary linked to this article.

## Online content

Any methods, additional references, Nature Portfolio reporting summaries, source data, extended data, supplementary information, acknowledgements, peer review information; details of author contributions and competing interests; and statements of data and code availability are available at 10.1038/s41586-025-08958-4.

## Supplementary information

Reporting Summary

## Figures and Tables

**Extended Data Table 1:**
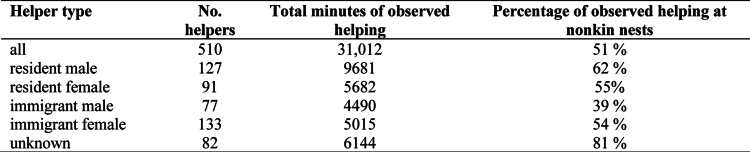
Proportion of helping time at nonkin nests

Nonkin nests are defined as those where the helper’s relatedness to both parents is either 0 for pedigree-based estimates (residents) or less than 0.125 for marker-based relatedness (immigrants and unknown). Cases where this relatedness value was unknown (2.6% of daily helping rates) were excluded.

**Extended Data Table 2:**
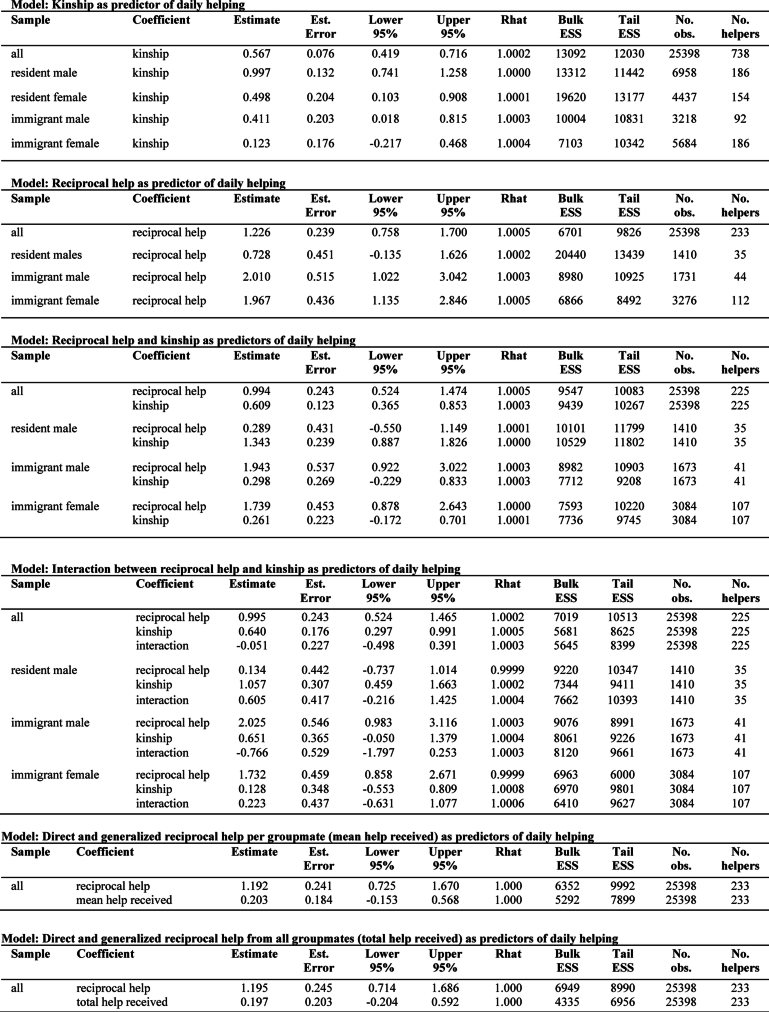
Models and Coefficients

Estimated regression coefficients from *brms* R package are shown for each sample of individuals with standard error (Est. Error), 95% Bayesian Credible Intervals (Lower 95%, Upper 95%), diagnostics for convergence including Rhat (4 chains), Bulk Effective Sample Size (ESS), Tail ESS, number of samples of possible daily helping across possible helpers (No. obs.), and number of helpers (No. helpers). ‘All’ category includes helpers of unknown dispersal history.

## Data Availability

All data are available via GitHub at https://github.com/AlexisDEarl/reciprocity_and_nepotism_in_superb_starlings/tree/main/data. The files with the raw results are also available via GitHub at https://github.com/AlexisDEarl/reciprocity_and_nepotism_in_superb_starlings/tree/main/results.

## Code availability

The code used in this paper is available via GitHub at https://github.com/AlexisDEarl/reciprocity_and_nepotism_in_superb_starlings/tree/main/code.
