# Supplementary material for: A cryptic role for reciprocal helping in a cooperatively breeding bird
Source: Nature. 2025 May 7;642(8067):381–8. doi: 10.1038/s41586-025-08958-4 (PMC12158779; doi:10.1038/s41586-025-08958-4)
Supplement: Supplementary file 1 — Reporting Summary [file 41586_2025_8958_MOESM1_ESM.pdf]

nature portfolio

Corresponding author(s): Alexis D. Earl, Gerald G. Carter, Dustin R. Rubenstein

Last updated by author(s): Jan 16, 2025

Reporting Summary

Nature Portfolio wishes to improve the reproducibility of the work that we publish. This form provides structure for consistency and transparency in reporting. For further information on Nature Portfolio policies, see our Editorial Policies and the Editorial Policy Checklist.

Statistics

For all statistical analyses, confirm that the following items are present in the figure legend, table legend, main text, or Methods section.

n/a	Confirmed	
☐	☒	The exact sample size (*n*) for each experimental group/condition, given as a discrete number and unit of measurement
☒	☐	A statement on whether measurements were taken from distinct samples or whether the same sample was measured repeatedly
☐	☒	The statistical test(s) used AND whether they are one- or two-sided<br>*Only common tests should be described solely by name; describe more complex techniques in the Methods section.*
☐	☒	A description of all covariates tested
☐	☒	A description of any assumptions or corrections, such as tests of normality and adjustment for multiple comparisons
☐	☒	A full description of the statistical parameters including central tendency (e.g. means) or other basic estimates (e.g. regression coefficient) AND variation (e.g. standard deviation) or associated estimates of uncertainty (e.g. confidence intervals)
☐	☒	For null hypothesis testing, the test statistic (e.g. *F*, *t*, *r*) with confidence intervals, effect sizes, degrees of freedom and *P* value noted<br>*Give P values as exact values whenever suitable.*
☐	☒	For Bayesian analysis, information on the choice of priors and Markov chain Monte Carlo settings
☐	☒	For hierarchical and complex designs, identification of the appropriate level for tests and full reporting of outcomes
☐	☒	Estimates of effect sizes (e.g. Cohen's *d*, Pearson's *r*), indicating how they were calculated

Our web collection on statistics for biologists contains articles on many of the points above.

Software and code

Policy information about availability of computer code

Data collection	We did not use code to collect data, only to analyze data. Data were collected in the field.
Data analysis	All statistical analyses were done in R version 4.3.1 (R Core Team 2023).

For manuscripts utilizing custom algorithms or software that are central to the research but not yet described in published literature, software must be made available to editors and reviewers. We strongly encourage code deposition in a community repository (e.g. GitHub). See the Nature Portfolio guidelines for submitting code & software for further information.

Data

Policy information about availability of data

All manuscripts must include a data availability statement. This statement should provide the following information, where applicable:

- Accession codes, unique identifiers, or web links for publicly available datasets
- A description of any restrictions on data availability
- For clinical datasets or third party data, please ensure that the statement adheres to our policy

All data, code, and materials are available on GitHub: https://github.com/AlexisDEarl/reciprocity_and_nepotism_in_superb_starlings

Research involving human participants, their data, or biological material

Policy information about studies with human participants or human data. See also policy information about sex, gender (identity/presentation), and sexual orientation and race, ethnicity and racism.

Reporting on sex and gender	N/A
Reporting on race, ethnicity, or other socially relevant groupings	N/A
Population characteristics	N/A
Recruitment	N/A
Ethics oversight	N/A

Note that full information on the approval of the study protocol must also be provided in the manuscript.

Field-specific reporting

Please select the one below that is the best fit for your research. If you are not sure, read the appropriate sections before making your selection.

☐ Life sciences ☐ Behavioural & social sciences ☒ Ecological, evolutionary & environmental sciences

For a reference copy of the document with all sections, see nature.com/documents/nr-reporting-summary-flat.pdf

Ecological, evolutionary & environmental sciences study design

All studies must disclose on these points even when the disclosure is negative.

Study description	This study began in 2001 to study the evolution of cooperative breeding behavior in superb starlings in Kenya.
Research sample	Nine social groups of superb starlings at the Mpala Research Centre in Laikipia, Kenya (n=1175 individuals)
Sampling strategy	All birds in the population were trapped, marked, and monitored throughout their lives.
Data collection	We conducted focal observations of nests during breeding using a spotting scope. For each individual observed within 30 m of the nest, we recorded their identity, group membership, times of arrival and departure, their breeding role (i.e., "helper", "breeder"), and whether they arrived with food, vocalized, or mobbed predators at the nest.
Timing and spatial scale	Banding of this population began in 2001 and focal observations began in 2002. This study includes data from 2001-2021. Focal observations were typically 120 min in length (median = 120 min, mean = 125 min, range = 3 to 240 min, 90% were at least 120 min, 99.5% were at least 60 min). Over 730 days from 2002 until 2021 (40 breeding seasons), we observed 410 nests in the nine social groups and analyzed 12,112 visits to the nest with complete data (out of 14,588 events). In total, we observed 563 individually banded superb starlings acting as helpers and 254 as breeders over the course of the 20-year study. Focal observations were conducted during the two annual breeding seasons (short rains breeding season: October-December; long rains breeding season: March-June).
Data exclusions	No data were excluded after cleaning.
Reproducibility	N/A
Randomization	All individuals in the population were included in the analysis.
Blinding	N/A

Did the study involve field work? ☒ Yes ☐ No

Field work, collection and transport

Field conditions	Birds were monitored continuously throughout the year from 2001 to 2021 in a range of conditions.
Location	We collected data from 2001-2021 at the Mpala Research Centre in Laikipia, Kenya (0° 17'N 37° 52'E).

Access & import/export	All research was approved by the Kenyan National Commission for Science, Technology and Innovation, the Kenyan National Environmental Management Authority, the Kenya Wildlife Service, the Kenyan Wildlife Research and Training Institute, and the Mpala Research Centre.
Disturbance	Birds were monitored at a distance with spotting scopes.

Reporting for specific materials, systems and methods

We require information from authors about some types of materials, experimental systems and methods used in many studies. Here, indicate whether each material, system or method listed is relevant to your study. If you are not sure if a list item applies to your research, read the appropriate section before selecting a response.

Materials & experimental systems

n/a	Involved in the study	
☒	☐	Antibodies
☒	☐	Eukaryotic cell lines
☒	☐	Palaeontology and archaeology
☐	☒	Animals and other organisms
☒	☐	Clinical data
☒	☐	Dual use research of concern
☒	☐	Plants

Methods

n/a	Involved in the study	
☒	☐	ChIP-seq
☒	☐	Flow cytometry
☒	☐	MRI-based neuroimaging

Animals and other research organisms

Policy information about studies involving animals; ARRIVE guidelines recommended for reporting animal research, and Sex and Gender in Research

Laboratory animals	N/A
Wild animals	We used baited pull-string traps (during the non-breeding season) or mist nets (at active nests during the breeding season) to capture individuals first observed in this population after immigration or fledging. We also banded individuals as hatchlings in the nest during the two annual breeding seasons (short rains breeding season: October-December; long rains breeding season: March-June). Each of the 1175 individuals in the population was marked with a numbered metal ring and a unique series of colored leg bands
Reporting on sex	Sex was verified genetically for all individuals using PCR primers as previously described for this species.
Field-collected samples	N/A
Ethics oversight	All research was approved by the Institutional Animal Care and Use Committee at Columbia University (IACUC protocol #AC-AAAW6451), as well as the Kenyan National Commission for Science, Technology and Innovation, the Kenyan National Environmental Management Authority, the Kenya Wildlife Service, the Kenyan Wildlife Research and Training Institute, and the Mpala Research Centre.

Note that full information on the approval of the study protocol must also be provided in the manuscript.

Plants

Seed stocks	N/A
Novel plant genotypes	N/A
Authentication	N/A
